# The optimal aortic cross-clamp time in minimally invasive mitral valve surgery: still a conundrum?

**DOI:** 10.1093/ejcts/ezad215

**Published:** 2023-05-31

**Authors:** W Randolph Chitwood

**Affiliations:** Department of Cardiovascular Diseases, East Carolina Heart Institute, Brody School of Medicine, East Carolina University, Greenville, NC, USA

**Keywords:** Cross-clamp, Mortality, Minimally invasive, Mitral surgery

The deleterious myocardial effects of long periods of aortic cross-clamping have been concerning since the mid-1970s. Moreover, in the presence of ventricular dysfunction, both early- and long-term recovery are in greatest jeopardy. In the present article, by Doenst *et al.* [1], entitled *Aortic Cross-clamp Time Correlates with Mortality in the Mini-mitral International Registr*y, the authors retrospectively analysed 6878 minimally invasive mitral operations (MIMVS) done between 2015 and 2021 from 17 international heart centres. Of these, 75% were mitral repairs and 25% were replacements. Individual cohorts included minimal access using direct, video-assisted, or totally endoscopic/robotic visualization. For all groups, the authors examined the relationship between aortic cross-clamp time (ACXT) and mortality. For all groups, the 30-day mortality was 1.6%. The median ACXT for these operations was 85 min with the shortest for the direct visualization cohort (73 min) and longest for the robotic group (129 min). By the univariate analysis, they found that irrespective of visualization method, type of mini-access or cardioplegia regimen, mortality and morbidity (low cardiac output, acute kidney injury) were not higher with ACXTs of 60 min or somewhat longer (pNS, odds ratio = 1.4). However, when this time was increased to 90 or 120 min, mortality was significantly higher (*P* ≤ 0.001, odds ratio = 1.9 and 3.0, respectively). Of all operations, 25.4% had ACXTs of >120 min. Risk factors for death emerged as age, emergent surgery and low ejection fraction. Concomitant procedures did not prove to be an independent risk factor for these patients. Thus, this study suggests that there is less mortality for MIMVS when ACXTs are kept between 60 and 90 min.

In a prospective study, Ruggieri *et al.* [2] showed in propensity-matched coronary surgery patients that ACXTs >75 min were associated with a significantly higher 30-day mortality. In low- and high-risk patients, Al-Sarraf reported that in patients having various cardiac operations, the risk of mortality increased after an ACXT longer than 60 min and was highest after 90 min [3]. Swinkels *et al.* showed that with a sternotomy (STR) in aortic valve replacement patients that the mortality was higher if the ACXT was longer than 60 min [4]. Lange and group compared a propensity-matched cohort of MIMVS operations (ACXT = 86 min) to those with a conventional STR where the mean ACXT was 74 min. The overall mortality was statistically the same despite the 12-min longer MIMVS arrest times [5]. Pojar *et al.* [6] reported a propensity-matched study where the MIMVS and STR cohorts had ACXTs of 98 and 85 min, respectively. There was no statistical difference (pNS) in 30-day mortality (MIMVS = 1% vs STR = 3%) A meta-analysis by Cheng *et al.* of 24 individual cohort studies compared MIMVS patients to STR ones [7]. Cross-clamp times for the MIMVS varied from 47 to 136 min with the STR group times less and between 24 and 110 min. The 30-day mortality was 0.9% versus 1.3% (pNS) despite significantly increased ACXTs (95 vs 74 min). An earlier meta-analysis by Modi *et al.* also showed equal mortality despite significantly longer ACXTs for the MIMVS cohort [8].

Most recently, Mori *et al.* [9] reviewed data from the Society of Thoracic Surgeons Adult Cardiac Surgery Database for 61 322 patients having undergone a mitral repair between 2015 and 2021. The overall operative mortality was 0.7%, 1.1% and 1.2% for robotic, min-thoracotomy and sternotomy patients, respectively (pNS). Many of these patients had adjunctive procedures such as tricuspid repair and atrial fibrillation ablations. They found that mortality and morbidity were lower in high-volume centres with the threshold being 40 cases over the study period and declined even more for centres doing over 200 cases. Propensity score matching identified 5540 pairs of robotic (ROB) versus minithoracotomy and 6962 pairs of robotic versus sternotomy (STR) patients. In the ROB versus minithoracotomy cohort, patient ages (61 vs 62 years), ACXTs (95 vs 94 min), mortality (0.8% vs 0.8%), and morbidity (6.7% vs 6.0%) all were statistically equal. In the ROB versus STER group, patient ages (61 vs 61 years), ACXTs (95 vs 94 min), mortality (0.7% vs 0.6%) and morbidity (6.2% vs 6.2%) were also the same.

Minimally invasive mitral surgery has challenges. As shown above often have significantly longer ACXTs than operating through an STR. Limited access requires more attention to myocardial protection. An STR avails the possibility of topical cooling as well as better ventricular decompression and deairing. Also, MIMVS operations require more operator skill, which is acquired best with larger case volumes. Conversely, most of these patients are <65 years old and have preoperative normal ventricular function with less preoperative co-morbidities. Additionally, they usually are a first-time operation and are not an emergency. From these studies, the consensus seems that the safe ACXT is 60–90 min with mortality rising significantly after 120 min. This is what Doenst *et al.* determined in their current study [1]. The report by Mori and Geirsson is encouraging as it showed statistically equal ACXTs (95 min) with MIMVS, ROB and STR, all of which had an equally low mortality. Thus, less-invasive mitral valve surgery, whether using direct, endoscopic, or robotic visualization, continues to emerge with many benefits in Europe and the USA as being safe and effective with excellent long-term outcomes. To this end, these reports have helped surgeons to unwind the ‘Gordian Knot Conundrum’ of aortic cross-clamp time versus operative mortality and morbidity.
